# Inhibition of eEF2K synergizes with glutaminase inhibitors or 4EBP1 depletion to suppress growth of triple-negative breast cancer cells

**DOI:** 10.1038/s41598-021-88816-1

**Published:** 2021-04-28

**Authors:** YoungJun Ju, Yaacov Ben-David, Daniela Rotin, Eldad Zacksenhaus

**Affiliations:** 1 Max Bell Research Centre, Toronto General Research Institute, University Health Network, 101 College Street, Suite 5R406, Toronto, ON M5G 1L7 Canada; 2 The Key Laboratory of Chemistry for Natural Products of Guizhou Province, Chinese Academic of Sciences, Guiyang, 550014 Guizhou China; 3 State Key Laboratory for Functions and Applications of Medicinal Plants, Guizhou Medical University, Guiyang, 550025 China; 4 Program in Cell Biology, The Peter Gilgan Center for Research and Learning, The Hospital for Sick Children, Toronto, ON Canada

**Keywords:** Cancer, Breast cancer

## Abstract

The eukaryotic elongation factor-2 kinase, eEF2K, which restricts protein translation elongation, has been identified as a potential therapeutic target for diverse types of malignancies including triple negative breast cancer (TNBC). However, the contexts in which eEF2K inhibition is essential in TNBC and its consequences on the proteome are largely unknown. Here we show that genetic or pharmacological inhibition of *eEF2K* cooperated with glutamine (Gln) starvation, and synergized with glutaminase (GLS1) inhibitors to suppress growth of diverse TNBC cell lines. eEF2K inhibition also synergized with depletion of eukaryotic translation initiation factor 4E-binding protein 1 (eIF4EBP1; 4EBP1), a suppressor of eukaryotic protein translation initiation factor 4E (eIF4E), to induce c-MYC and Cyclin D1 expression, yet attenuate growth of TNBC cells. Proteomic analysis revealed that whereas eEF2K depletion alone uniquely induced Cyclin Dependent Kinase 1 (CDK1) and 6 (CDK6), combined depletion of eEF2K and 4EBP1 resulted in overlapping effects on the proteome, with the highest impact on the ‘Collagen containing extracellular matrix’ pathway (e.g. COL1A1), as well as the amino-acid transporter, SLC7A5/LAT1, suggesting a regulatory loop via mTORC1. In addition, combined depletion of eEF2K and 4EBP1 indirectly reduced the levels of IFN-dependent innate immune response-related factors. Thus, eEF2K inhibition triggers cell cycle arrest/death under unfavourable metabolic conditions such as Gln-starvation/GLS1 inhibition or 4EBP1 depletion, uncovering new therapeutic avenues for TNBC and underscoring a pressing need for clinically relevant eEF2K inhibitors.

## Introduction

Triple negative breast cancer (TNBC) is a heterogeneous disease characterized by reduced expression of the Estrogen (ERα) and Progesterone receptors and by lack of amplification of the *HER2/ERRB2/NEU* oncogene^1,2^. TNBC comprises at least 6 different subtypes that can be further stratified on the basis of distinct oncogenic alterations^3,4^. Conventional treatment is based on cytotoxic chemotherapy, which is effective for approximately 20% of patients, but metastatic disease is lethal with a median survival rate of 1 year. Recent combination therapies of antineoplastic drugs with immune-check point blockade inhibitors such as anti-PD-L1^5^ extend life span, but patients eventually succumb to the disease, exposing a pressing need for novel approaches.

Through analysis of a mouse model of *PTEN/TP53*-deficient TNBC, we previously identified eukaryotic elongation factor-2 kinase (eEF2K) as a potential target for therapeutic intervention^6^. eEF2K, also known as calmodulin-dependent protein kinase III (CAMKIII), is a cytosolic threonine kinase that regulates protein synthesis by phosphorylating the elongation factor eEF2 at T56^7^. This phosphorylation blocks eEF2 ability to bind ribosomes, thus reducing protein synthesis. Inhibition of eEF2K promotes excessive protein translation elongation and is thought to “push cells over-the cliff” under stress conditions such as nutrient deprivation, or in tumor cells in which high PI3K/mTOR signaling or MYC amplification promote protein translation^8–10^.

The observation that eEF2K inhibition is most effective under stress such as nutrient deprivation raised the question of whether molecular inhibition of specific cellular proteins would cooperate with eEF2K antagonists to accelerate cell demise even under non-stressful/nutrient limiting conditions. Here, we demonstrate that eEF2K inhibitors synergize with glutaminase (GLS1) inhibitors to suppress growth of diverse TNBC cell lines. Moreover, combined inhibition of eEF2K and the protein translation initiation inhibitor, 4EBP1, synergizes to suppress cancer growth, suggesting that excessive protein translation or induction of incompatible signalling pathways is lethal. Finally, using mass spectrometry, we observed common and distinct proteins that are induced or repressed following eEF2K and/or 4EBP1 depletion. Together, our results identify novel vulnerabilities and corresponding combination therapies for TNBC and highlight the need for the development of medicinal inhibitors for this protein translation elongation factor.

## Results

### Pharmacological inhibition of eEF2K cooperates with glutamine deprivation or glutaminase (GLS1) inhibitors to suppress breast cancer cell growth

**Figure 1:**
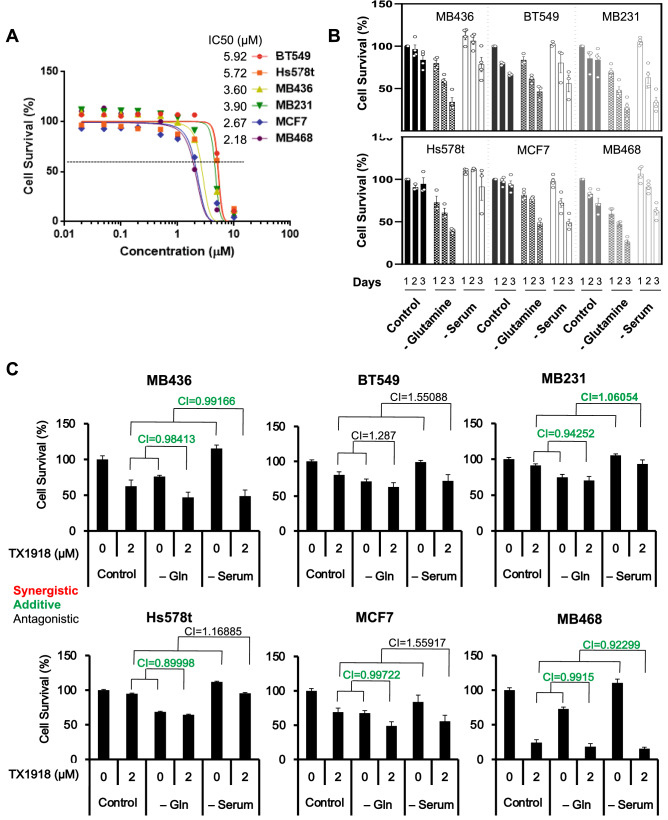
Pharmacological inhibition of eEF2K suppresses growth of breast cancer cell lines under nutrient deprivation (ND). (**A**) Dose response curves for the eEF2K inhibitor TX1918 of indicated breast cancer cell lines using MTT assays. IC50 for each line is indicated (see Supplementary Fig. S1A for details). (**B**) Time-dependent response of indicated breast cancer cell lines to TX1918 (0 or 2 µM) in normal medium (control), without serum or without glutamine (Gln). Numbers in brackets denote incubation periods in days. (**C**) Synergistic effects of TX1918 under serum or Gln deprivation, determined by CompuSyn analysis. Combination Index: CI < 0.85 denotes synergy (red), 0.85 < CI < 1.1, additive (Green), and CI > 1.1, antagonistic effect (black).

To determine the consequences and contexts in which eEF2K inhibition restricts cell growth, we first determined the effect of the eEF2K inhibitor TX1918^11^ on aggressive triple breast cancer (TNBC) cell lines (BT549, Hs578t, MDA-MB-436, MDA-MB-231, MDA-MB-468) as well as the luminal breast cancer line MCF7, cultured in complete media (DMEM) supplemented with 10% fetal calf serum (FCS; Fig. 1A). All these lines exhibited a similar drug response curves though with different sensitivity, with MDA-MB-468 being most responsive (IC50 = 2.18 µM) and BT549 most resistant (IC50 = 5.92 µM). A dot plot and statistical analysis of the IC50s for each line are shown in Supplementary Figure S1A. We then assessed the effect of nutrient-deprivation (ND) on sensitivity to TX1918, using two conditions: serum deprivation (0% FCS) or glutamine (Gln) starvation. In these experiments, cells were treated with TX1918 for 3 days, the last day of which was under serum- or Gln-deprivation (for 1 day). Cells were much more sensitive to eEF2K inhibition in the absence of Gln than in the absence of serum (Fig. 1B). CompuSyn software was used to calculate synergy with a combination index CI < 0.85 denoting synergy, 0.85 < CI < 1.1 additive and CI > 1.1 antagonistic effect [http://www.combosyn.com]^12^. With the exception of BT549 cells, all other lines exhibited additive effects between TX1918 and Gln starvation but not with serum deprivation (Fig. 1C).

**Figure 2:**
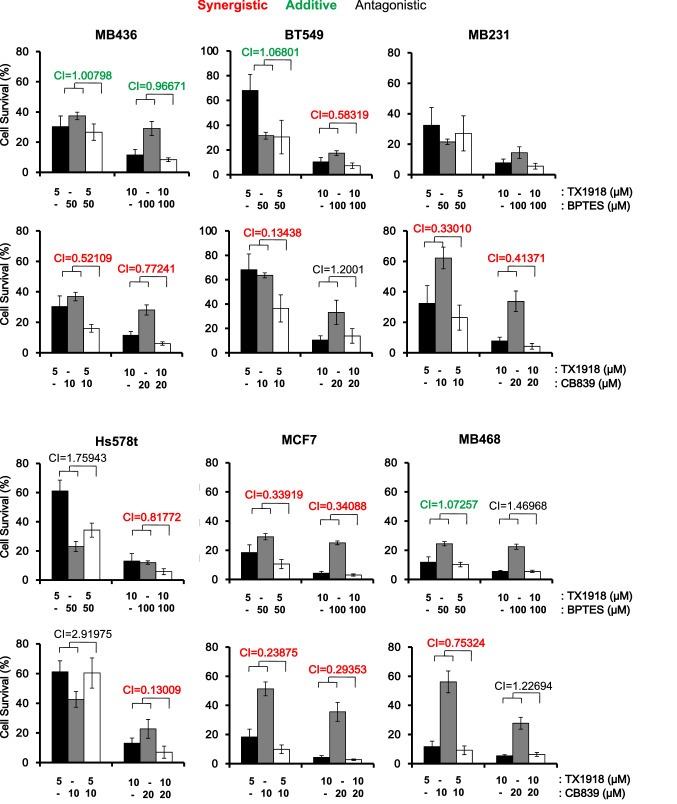
Synergistic inhibition of cell growth by combined treatment with the eEF2K inhibitor TX1918 plus Glutaminase 1 (GLS1) inhibitors (CB-839; BPTES). Breast cancer cells were treated with indicated concentrations of the eEF2K inhibitor TX1918, and the GLS1 inhibitors BPTES or CB-839, and effects on cell growth were determined by MTT assays. Combination Index (CI) was calculated by Compusyn software; synergistic effect (CI < 0.85; red), additive (0.85 < CI < 1.1; green), and antagonistic (CI > 1.1; black).

Gln is deaminated by glutaminase (GLS) to glutamate, which then enters the TCA cycle in the mitochondria or is directly used for anabolic metabolism^13,14^. We next asked whether inhibition of Gln usage by GLS1 inhibitors would also cooperate with TX1918. BPTES and CB-839 (telaglenastat)^15^ are selective, non-competitive inhibitors that target GLS1 but not GLS2. Remarkably, all tested TNBC cell lines exhibited dramatic and synergistic sensitivity to at least one combination of TX1918 plus BPTES or CB-839 (Fig. 2). Notably, the level of inhibition we observed with each drug alone is consistent with published reports using pyruvate-containing DMEM media, used herein, which attenuate sensitivity to GLS inhibition^6,16–21^. In addition, unlike other luminal breast cancer lines, MCF7 cells are highly sensitive to CB-839 (Fig. 2)^15^. These results offer a potential new modality for metabolic targeting of diverse types of TNBCs, using eEF2K-based therapy, and highlight the need for clinically relevant inhibitors for this protein kinase.

### Combined genetic silencing of protein translation elongation (eEF2K) and protein translation initiation (eIF4EBP1) inhibitors cooperates to induce CyclinD1 and c-MYC protein expression and suppress breast cancer cell growth

One possible explanation for the aforementioned synergy is that combined eEF2K and GLS1 inhibitors push cells “over the cliff” by forcing them (via eEF2K inhibition) to synthesize proteins under conditions in which cells are deprived of glutamate, an amino-acid that provides a nitrogen backbone for metabolic anabolism via the TCA cycle^14^. We therefore asked whether further acceleration of protein synthesis would also synergize with eEF2K inhibition to restrict cell growth by forcing cells to synthesize proteins beyond their capacity. To examine this possibility, we knocked-down eEF2K together with eukaryotic translation initiation factor 4E-binding protein 1 (eIF4EBP1, also known as 4EBP1)^22^. 4EBP1 is an allosteric inhibitor of eukaryotic translation initiation factor 4E (eIF4E), a component of the eIF4F complex, which together with eIF4A and eIF4G, binds “capped” (7-methyl-guanosine modified) mRNAs and directs them to ribosomes to initiate protein translation^23,24^. 4EBP1 and 4EBP2 share a conserved eIF4E binding motif and both are regulated by mTORC1, hence compensate for each other to some degree. Here, we asked whether disruption of 4EBP1 alone would sensitize cells to the effect of eEF2K loss.

**Figure 3:**
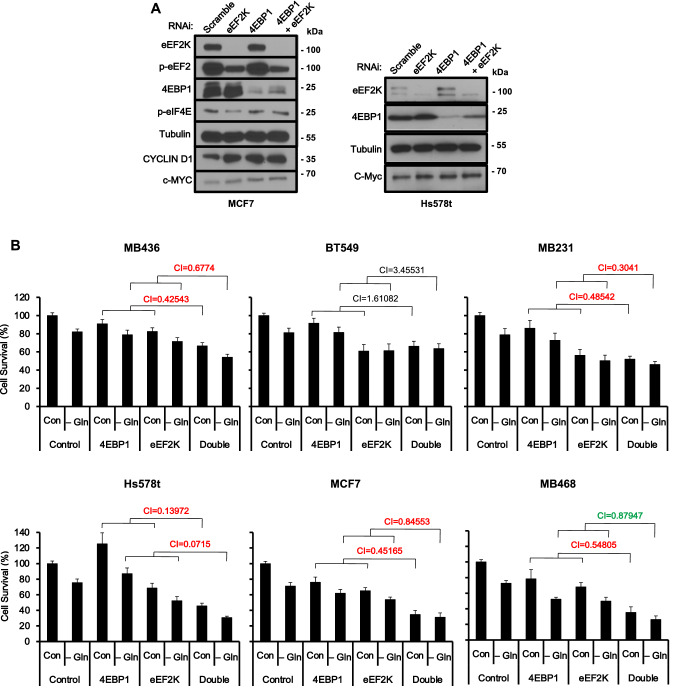
Induction of CYCLIN D1 and c-MYC and synergistic inhibition of breast cancer cell growth following combined knockdown of *eEF2K* and *4EBP1* under nutrient deprivation. (**A**) CYCLIN D1 and c-MYC expression following transient RNAi-mediated knockdown of *eEF2K* and/or *4EBP1* in MCF7 (left) and Hs578t (right) cells. (**B**) Cell growth following combined knockdown of *eEF2K* and *4EBP1* in indicated breast cancer cell lines under nutrient deprivation. CIs were analyzed by CompuSyn software: synergy (CI < 0.85; red), additive (0.85 < CI < 1.1; green), and antagonistic (CI > 1.1; black).

Effective depletion of eEF2K, 4EBP1 or both proteins in MCF7 cells by RNAi-mediated knockdown is demonstrated in Fig. 3A (un-cropped Western blots are shown in Supplementary Fig. S2). Phospho-T56-eEF2 (p-eEF2) was reduced by depletion of its kinase, eEF2K, but not by depletion of 4EBP1. Among known eIF4E regulated mRNAs are CYCLIN D1 and MYC^25–27^. Interestingly, transient depletion of either eEF2K or 4EBP1 in MCF7 cells induced CYCLIN D1 and cMYC protein expression level to similar extent (Fig. 3A). We note that in order to test for changes in protein levels, we used in these experiments identical volumes of protein lysates extracted from the same number of each RNAi-treated cells.

*eEF2K* and *4EBP1* were then transiently knocked-down in the different breast cancer cell lines and impact on growth was determined by MTT assay, which measures redox potential of viable mammalian cells (Fig. 3B). With the exception of BT549, there was a robust synergy between *eEF2K* and *4EBP1* knockdown, with the strongest synergy observed in the TNBC line Hs578t (CI = 0.0715). Thus, depletion of inhibitors of protein translation initiation (4EBP1) and elongation (eEF2K) synergizes to suppress growth of diverse TNBC cells; this suppression correlated with elevated expression of proteins associated with induction of cell proliferation, and was further potentiated under nutrient deprivation.

**Figure 4:**
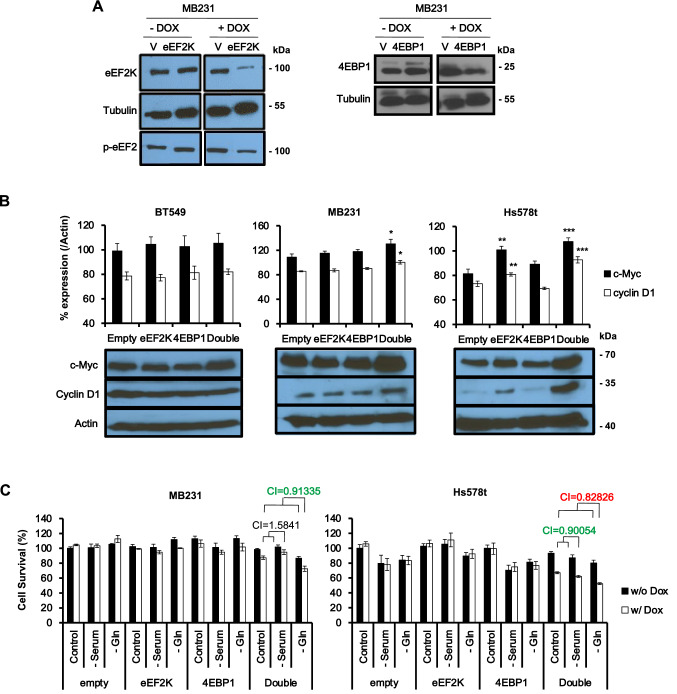
Establishment of tet-inducible knockdown of *eEF2K* and/or *4EBP1* in TNBC cell lines, and impact on cell growth. (**A**) Western blots showing the effect of tet-inducible *eEF2K* or *4EBP1* shRNAs on expression of the respective proteins in MDA-MB-231 cells. V denotes empty control vector. Uncropped images are shown in Supplementary Fig. S2. (**B**) Establishment of tet-inducible shRNA-mediated knockdown of *eEF2K* plus *4EBP1* in three different TNBC cell lines, and impact on CYCLIN D1 and c-MYC expression. *Denotes *P* < 0.05, **P < 0.01, and ***P < 0.001 versus empty control vector by ANOVA; n = 4. (**C**) Cell survival determined by MTT assays in tet-inducible *eEF2K*- and/or *4EBP1*-depleted MDA-MB-231 and HS578t cells under normal or nutrient deprivation conditions (lacking serum or Gln). CIs was calculated by Compusyn software; synergy (CI < 0.85; red), additive (0.85 < CI < 1.1; green), and antagonistic (CI > 1.1; black).

To investigate the cooperation between eEF2K and 4EBP1, we generated stable cell lines expressing pTRIPZ-tet-on inducible shRNAs directed against both mRNAs (Fig. 4A; Supplementary Fig. S1B). Doxycycline-induced shRNAs for both genes more efficiently depleted eEF2K than 4EBP1 and, as expected, was not as robust as that obtained following transient RNAi mediated silencing (Fig. 4A,B vs. Fig. 3A). Likewise, depletion of eEF2K resulted in reduced phosphorylation of its target p-eEF2, but again, was not as effective as following transient and more robust depletion of eEF2K by RNAi (Fig. 4A). Nevertheless, these stable lines allowed controlled, long-term inhibition of these protein translation initiation and elongation factors.

Next, we determined the effect of single or combined knockdown of eEF2K and/or 4EBP1 on expression of CYCLIN D1 and c-MYC in cell lines with low (BT549), moderate (MDA-MB-231) or high (Hs578t) sensitivity to transient silencing of *eEF2K* and *4EBP1* (Fig. 4B). Significant induction of CYCLIN D1 and c-MYC was seen in Hs578t and MDA-MB-231 cells, with only a trend but not statistically significant increase in levels of these proteins in BT549 cells following double eEF2K plus 4EBP1 depletion (Fig. 4B). The latter results are consistent with the relative resistance of BT549 cells to loss of *eEF2K* and *4EBP1*.

In addition to the MTT assay, we also assessed the effect of eEF2K and/or 4EBP1 depletion side-by-side with direct cell counting via trypan blue exclusion assays (Supplementary Fig. S1B–C). Both methods showed significant inhibition of cell growth following combined *eEF2K* plus *4EBP1* knockdown in DOX- versus no-DOX-treated cells, though the effect was more significant in the MTT assay. Importantly, combined depletion of eEF2K plus 4EBP1, and to a lesser extent, eEF2K depletion alone, significantly reduced relative overall cellular ATP levels (Supplementary Fig. S1D). Silencing of *eEF2K* plus *4EBP1* may exhaust cellular ATP levels due to excessive protein translation, and may thereby underlie the growth suppression observed following depletion of these factors.

Finally, we determined the effect of combined *eEF2K* plus *4EBP1* depletion on cell proliferation under normal, serum deprivation or Gln starvation. As noted, stable shRNA-mediated depletion of these factors led to more modest suppression of cell growth (Fig. 4C) relative to transient knockdown via RNAi (Fig. 3B), which is consistent with the reduced efficacy of knockdown. Nonetheless, in MDA-MB-231 and Hs578t TNBC cells, dox-inducible silencing of *eEF2K* plus *4EBP1* suppressed proliferation and further cooperated with Gln starvation to exert additive or synergistic inhibition of cell growth, respectively (Fig. 4C). We therefore used Hs578t cells with tet-inducible shRNA for *eEF2K* and/or *4EBP1* (Supplementary Fig. S1B), for subsequent proteomic assays.

### Identification of global changes in the proteome following *eEF2K* and/or *4EBP1* knock-down by LC–MS/MS

**Figure 5:**
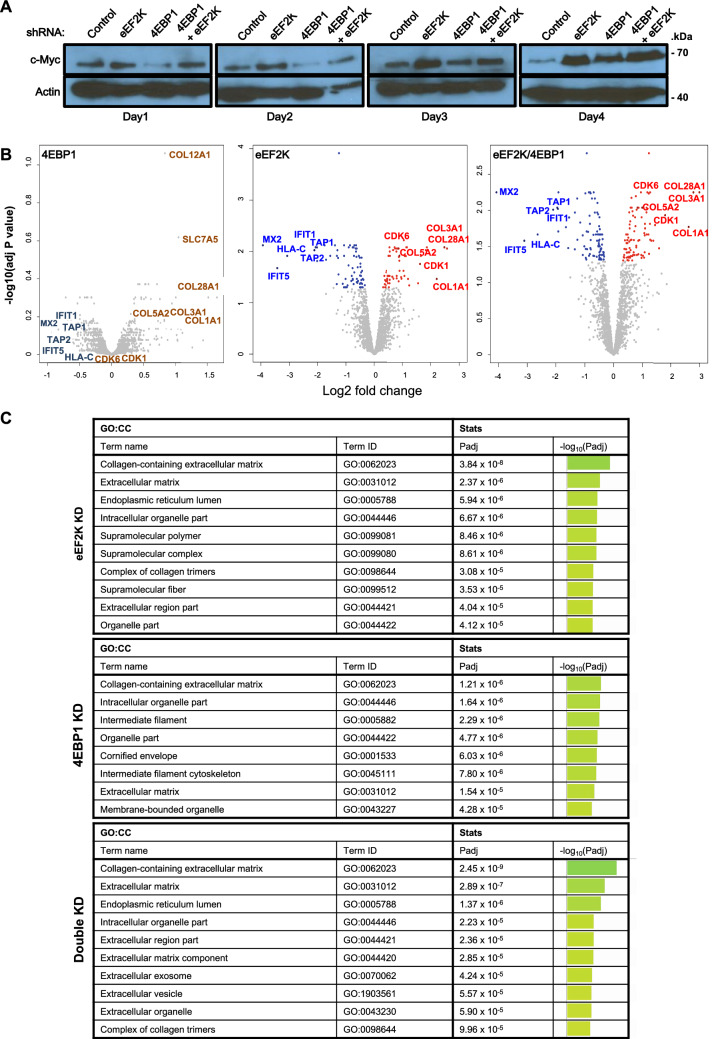
LC–MS/MS-based identification of proteins that are up-regulated following *eEF2K* and/or *4EBP1* knock-down in HS578T triple negative breast cancer cells. (**A**) Time-dependent induction of c-MYC in DOX-induced *eEF2K* and/or *4EBP1* shRNA expressing Hs578t cells under control conditions (normal medium) used for subsequent LC–MS/MS analysis. (**B**) Volcano plots of LC–MS/MS data following knockdown of *eEF2K*, *4EBP1* or both. Selected proteins are highlighted. Note the inhibition (left) or induction (right) of similar proteins, albeit at different significance, and more resemblance of the *eEF2K* and double knockdown than the *4EBP1* plots. For the latter, COL12A1 and SLC7A5 are also indicated. (**C**) Gene ontology analysis of proteins induced > 0.5 (Log2 fold change) after *eEF2K* and/or *4EBP1* knockdown relative to empty vector control. ‘Collagen containing extracellular matrix’ pathway is identified as a top pathway induced after *eEF2K* and/or *4EBP1* knockdown.

To determine the consequences of long-term depletion of eEF2K alone or together with 4EBP1 in Hs578t cells on the steady state level of the proteome, we used Liquid chromatography–mass spectrometry (LC–MS/MS) analysis (Orbitrap Fusion Lumos Tribrid). We first optimized conditions by following the expression of c-MYC after DOX-induced shRNA-mediated knockdown of *eEF2K*, *4EBP1* or both, over a four-day period under normal growth conditions. c-MYC expression was elevated by day 3 under all conditions and reached a plateau at day 4 (Fig. 5A). We then subjected independent triplicate lysates from each group to LC–MS/MS analysis and identified cellular proteins whose expression was induced or suppressed by at least 2 folds (Supplementary Tables S1 and S2). These proteins may be direct targets of eEF2K or 4EBP1, or indirect, downstream consequences of enhanced protein translation initiation and/or elongation.

To assess the distribution of the LC–MS/MS data, we performed Volcano plot analysis, which compares protein fold change as a function of adjusted_*P* values across all identified and quantified proteins^28^. Red and blue dotes denote statistically significant upregulated or downregulated proteins, respectively, whereas gray dotes indicate insignificant changes relative to the control group. Notably, adjusted_*P* values are much higher than *P* values, thus reducing significance quite dramatically as is seen with the 4EBP1 plot. Yet, the trend indicates substantial overlap between proteins induced following 4EBP1 and eEF2K depletion, some of which are highlighted (Fig. 5B; Supplementary Table S3). This trend is also observed in the double knockdown, in which the significance (lower adjusted_*P* values) of these overlapping proteins further increases. Notably, the *eEF2K* and *eEF2K*/*4EBP1* knockdown plots are more similar to each other than that of *4EBP1* knockdown alone. This is likely due to incomplete depletion of 4EBP1 and compensation by its homologue, 4EBP2.

One example of particular interest is the induction of Solute Carrier Family 7 Member 5 (SLC7A5)/LAT1, a transporter of large neutral amino acids, primarily leucine (Leu). In addition to generating Glu, Gln provides the driving force for Leu entry into cells via LAT1. In turn, Leu entry stimulates mTOR translocation to the lysosomal membrane and mTORC1 activation, a step critical for induction of protein synthesis^29^. While fold change for SLC7A5/LAT1 was low: 1.32 fold increase after 4EBP1 depletion; 1.28 after eEF2K depletion; and 1.35 following 4EBP1 plus eEF2K double depletion, it was the second most significantly elevated protein in the 4EBP1 volcano plot relative to control (*P* = 0.00022; adjusted_*P* value = 0.24). For eEF2K the effect on SLC7A5/LAT1 was more significant (*P* = 0.00065; adjusted_*P* value = 0.024), and for eEF2K/4EBP1, significance further increased (*P* = 0.00011; adjusted_*P* value = 0.0092). Thus, SLC7A5/LAT1 is regulated by cap-dependent protein translation initiation and elongation, establishing a regulatory loop in which neutral amino acid transport via LAT1 induces mTORC1, which in turn inhibits eEF2K and 4EBP1 leading to protein translation and synthesis of LAT1. Despite the only 1.3 fold increase in SLC7A5/LAT1 level, the results as indicated by the adjusted_*P* value is highly significant. However, we limited subsequent analysis to proteins that increased ≥ 2 folds. Notably, Cyclin D1 and MYC were not detected by our LC–MS/MS analysis, even-though they are significantly induced following eEF2K depletion in Hs578t cells (Fig. 4B), demonstrating the high stringency of the LC–MS/MS analysis.

To ask whether inhibition of eEF2K and/or 4EBP1 induces specific signalling pathways, we performed G:profiler on proteins induced > 0.5 (Log2 fold change) relative to empty vector control. Gene Ontology Cellular Component (GO-CC) analysis revealed that ‘Collagen containing extracellular matrix’ was the most induced pathway following *eEF2K* and/or *4EBP1* knockdown (Fig. 5C). The ‘Extracellular matrix’ pathway was second highest after *eEF2K* or *eEF2K* plus *4EBP1* knockdown, and seventh following *4EBP1* silencing alone.

As noted, comparison of proteins that were induced 2 folds or more revealed a substantial overlap between those induced by eEF2K and 4EBP1 depletion (Supplementary Table S3). Specifically, of the 50 proteins induced over twofold following 4EBP1 depletion, 32 (64%) overlapped with eEF2K depletion, and 28/50 (56%) overlapped with eEF2K/4EBP1 double depletion. 115 of 135 (85%) proteins induced following *eEF2K* knockdown overlapped with double depletion.

**Figure 6:**
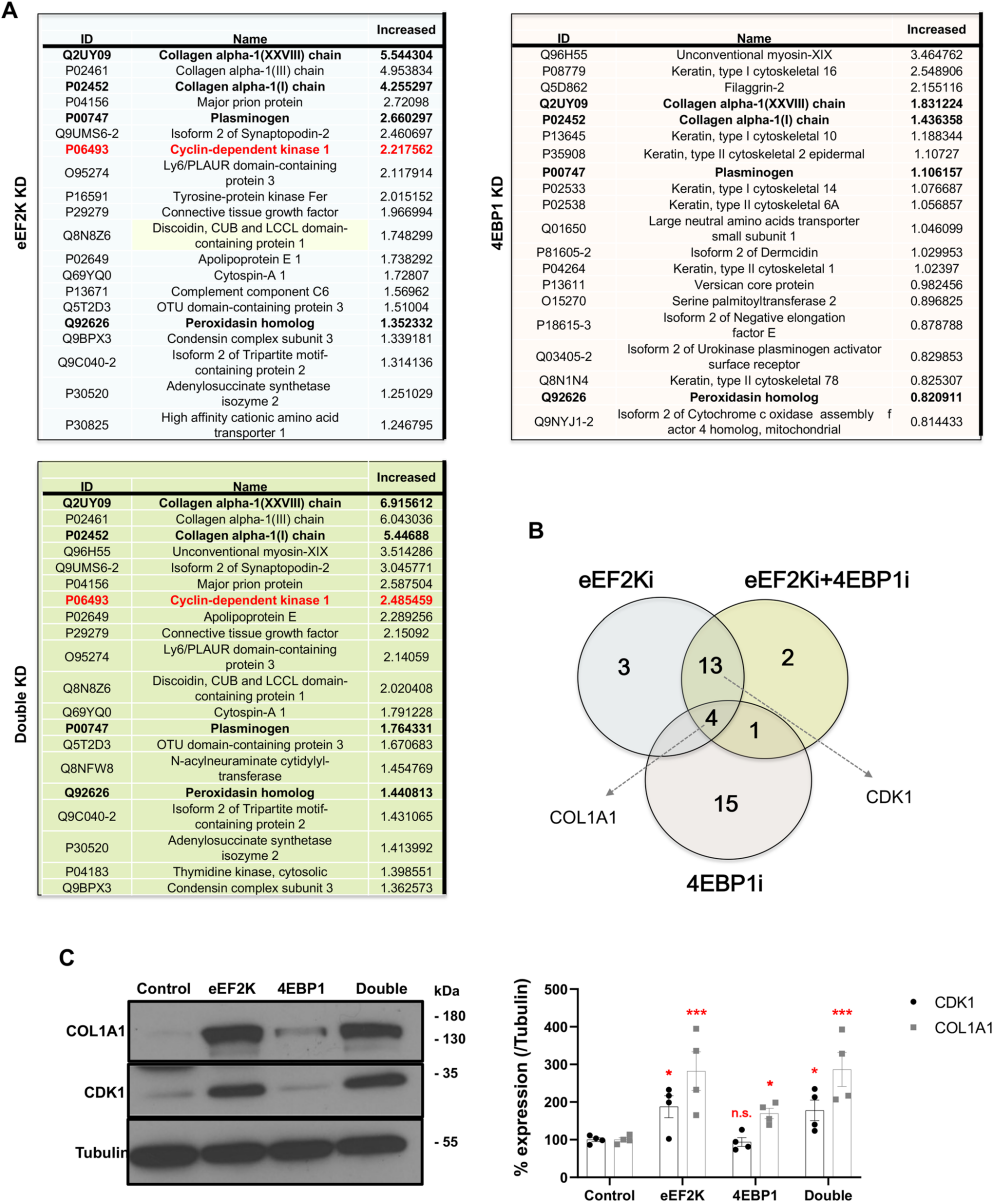
Induction and validation of COL1A1 and CDK1 following knockdown of *eEF2K* and/or *4EBP1*. (**A**) Top 20 up-regulated proteins from the LC–MS/MS analysis of *eEF2K* and/or *4EBP1* knocked-down (KD) HS578T cells. Common proteins are shown in bold. For full overlap of all proteins induced ≥ twofold following eEF2K and/or 4EBP1 depletion, see Supplementary Table S3. (**B**) Venn diagram showing the numbers of up-regulated proteins in the top 20 lists following *eEF2K* and/or *4EBP1* knockdown (i). COL1A1 (Collagen Type I Alpha 1 Chain) is elevated in all lines whereas CDK1 (Cyclin dependent kinase 1) is elevated following *eEF2K* but not *4EBP1* knockdown. (**C**) Western blot analysis showing that in accordance with the MS analysis, both COL1A1 and CDK1 proteins are significantly induced following *eEF2K* knockdown via RNAi, but only COL1A1 is significantly induced following 4EBP1 depletion in Hs578t cells. Left, a representative Western blot. Right, dot plot quantification of four independent experiments following *eEF2K* and/or *4EBP1* silencing by RNAi. *Denotes *P* < 0.05; **P < 0.01; ***P < 0.001 versus control by ANOVA; n.s. not significant; n = 4.

Figure 6A depicts the top 20 proteins induced 2 folds or more following knockdown of *eEF2K*, *4EBP1* or both protein translation inhibitors. In accordance with the GO-CC analysis, multiple collagens, such as collagen alpha-1 (XXVIII) chain (COL28A1) and COL1A1, were at the top of all three groups. Induction of COL3A1, COL28A1, COL5A2 and COL1A1 is highlighted in the volcano plots (Fig. 5B). Venn diagram of the top 20 most induced proteins revealed that four: COL28A1, COL1A1, PLG and PXDN, were shared by all three groups (Fig. 6B). Collagen chain components including COL28A1 are up-regulated in a transcriptional signature induced by a metastasis-promoting c-Src mutant in human breast cells^30^. In addition, COL1A1 promotes cell migration in vitro and metastasis in colorectal cancer by regulating the WNT/PCP pathway^31^. The plasminogen (PLG) activator/plasmin system is an enzymatic cascade that promotes matrix degradation and cell invasion, whereas Peroxidasin (PXDN) is a heme-containing peroxidase secreted into the extracellular matrix to modulate extracellular matrix formation.

In addition, one protein (MYP19) was observed in both the *4EBP1* and *eEF2K/4EBP1* knockdown groups, whereas 13 proteins (COL3A1, SYNPO2-2, PRNP, CDK1, APOE, CCN2, LYPD3, DCBLD1, SPECC1L, OTUD3, TRIM2-2, ADSS2, and NCAPG) were induced following eEF2K or eEF2K plus 4EBP1 depletion (Fig. 6B). Of the latter 13 proteins induced by eEF2K loss, SYNPO2 (Synaptopodin 2, Myopodin)^32^; LYPD3 (LY6/PLAUR Domain Containing 3)^33,34^; and SPECC1L (Sperm Antigen with Calponin homology and Coiled-Coil domains 1 Like)^35^, are implicated in actin-cytoskeleton reorganization and cell migration. Thus, a major pathway induced by eEF2K and 4EBP1 loss involves cell migration.

Induction of cell migration-related proteins following eEF2K depletion was previously found by MS analysis of a human lung carcinoma cell line (A549)^36^. That study also demonstrated functional increase in cell migration in response to eEF2K silencing. The migration-related proteins included ACTN4 (actinin-a4), ITGA2 (integrin a2), ITGA3 (integrin a3), ITGA4 (integrin a4), LAMB4 (laminin subunit b4) and LAMC1 (laminin subunit g1), as well as COL2A1 (collagen type II, a1). Importantly, of these proteins, ACTN4, ITGA2, and ITGA3 were also elevated in our LC–MS analysis (Supplementary Tables S1–2). In addition, related proteins such as ITGB1, ITGB3, ITGB5, ITGA11, LAMB1, LAMB2, LAMB3, and 10 different collagen genes (e.g. COL4A1, COL6A1) were induced following both eEF2K and 4EBP1 depletion in TNBC cells.

Thus, in two independent MS studies, using two cell lines representing different malignancies, effect on cell migration is likely a major outcome of eEF2K inhibition. Our study shows that many of these factors are also preferentially induced following 4EBP1 depletion, suggesting that these two factors, eEF2K and 4EBP1, can be exploited by upstream regulatory pathways to promote cell migration and invasion.

A second major class of targets of these regulators of protein translation are cell cycle proteins such as the previously reported c-MYC and CyclinD1 (Figs. 3, 4), as well as CDK1 (cyclin-dependent kinase 1), the only essential CDK in mammalian cells^37^, and the G1 cyclin D dependent kinase, CDK6 (Fig. 5B), both uniquely identified in the eEF2K-depleted LC–MS analysis. To validate these MS findings, we assessed expression of two representative proteins, COL1A1 and CDK1. COL1A1 was robustly and significantly induced by silencing *eEF2K* and, albeit to a lesser extent, *4EBP1* (Fig. 6C). In contrast, but in accordance with the MS analysis, CDK1 was specifically induced following eEF2K but not 4EBP1 depletion. Both CDK1 and COL1A1 are often amplified in breast cancer and high CDK1 mRNA expression significantly correlates with poor prognosis in breast cancer, with trend but not significant effects of both genes on survival of TNBC, possibly due to a smaller number of TNBC patients (Supplementary Fig. S3B–C).

The LC–MS analysis also identified proteins whose expression was reduced, rather than elevated, following knockdown of *eEF2K* and/or *4EBP1* (Fig. 5B; Supplementary Figure S4; Supplementary Table S2). The top three proteins suppressed by *eEF2K* knockdown were Interferon-induced GTP-binding protein MX2, Interferon-induced protein with tetratricopeptide repeats 3 (IFIT3), and HLA class I histocompatibility antigen, CW-17 alpha chain. The top three proteins suppressed by *4EBP1* knocked-down were RABEP1 (Rab GTPase-binding effector protein 1), SPAG5 (Sperm-associated antigen 5), and THD7A (Thrombospondin type-1 domain-containing protein 7A). The two IFN-induced proteins MX2 and IFIT3 as well as Interferon-induced protein with tetratricopeptide repeats 1 (IFIT1) were also suppressed by eEF2K and/or 4EBP1 depletion (Fig. 5B; Supplementary Fig. S4).

Low expression of these proteins is likely an indirect consequence of induction of upstream transcriptional or post-transcriptional/post-translational repressors that are regulated by eEF2K and 4EBP1. Interestingly, eEF2K depletion induced Otu Deubiquitinase, OTUD3. Its paralog, OTUD5, was shown to suppress type I IFN-dependent innate immune response by cleaving the polyubiquitin chain from an essential type I interferon adaptor protein^38^. Whether OTUD3 has similar function in shutting down IFN-dependent immune response is unknown. These effects may point to a suicidal pathway that is triggered by over-induction of 4EBP1 and eEF2K regulated protein synthesis, leading to inhibition of the innate immune response in preparation for cell demise.

Together, the LC–MS analysis uncovered long-term consequences of induction of protein translation initiation (4EBP1-depletion) and elongation (eEF2K-depletion), revealing overlapping as well as unique downstream cellular targets.

## Discussion

**Figure 7:**
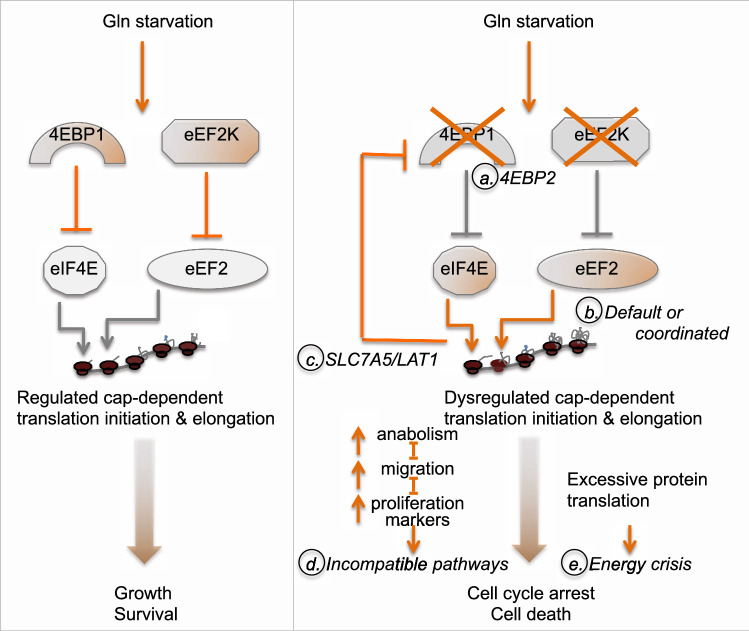
A working model for the effect of eEF2K and 4EBP1 on protein translation and cell growth/survival. Left, under glutamine starvation, eEF2K and 4EBP1 attenuate protein translation allowing cells to adapt to nutrient deprivation without loss of viability. Right, eEF2K and 4EBP1 inhibition enforces enhanced protein translation initiation and elongation, leading to cell cycle arrest or death. The model raises several questions and predictions (a–e)—see text for details.

We demonstrate herein that pharmacological or genetic inhibition of the eukaryotic elongation factor-2 kinase, eEF2K, cooperates with glutamine-deprivation and synergizes with glutaminase (GLS1) inhibition, as well as with depletion of eukaryotic translation initiation factor 4E-binding protein 1 (4EBP1) to suppress the growth of highly aggressive TNBC cell lines in vitro. Additive effects were seen between eEF2K and glutamine-starvation in 4 of 5 different TNBC cells, as well as in luminal MCF7 breast cancer cells. Potent synergy was observed between eEF2K and GLS1 inhibitors in all 5 lines, pointing to glutamate, the production of which is catalyzed by GLS1 from Gln, as the limiting factor. Glutamate provides carbon–nitrogen backbone for synthesis of amino-acids such as proline, arginine and α-ketoglutarate^14^. The latter is incorporated into the TCA cycle, which produces metabolites required for anabolic metabolism as well as the regeneration of NADH, subsequently used for ATP production via oxidative phosphorylation^39^. Thus, GLS1 inhibition restricts levels of amino-acids and ATP required for protein synthesis. We propose a model in which the combination of eEF2K inhibition, which consumes such metabolites, and their depletion by GLS1 inhibitors, leads to a metabolic collapse and cell demise (Fig. 7).

This model is supported by our observation that combined inhibition of eEF2K plus 4EBP1 also synergizes to kill TNBC cells. Possibly, combined increased in 5′ capped mRNA translation initiation and elongation accelerates metabolic failure and cell death. Alternatively, specific proteins such as CyclinD1 and c-MYC as well as CDK1 and CDK6 identified herein by Mass Spectrometry analysis, may induce death by pushing cells over the cliff when deprived of sufficient glutamine to sustain metabolic demand. Notably, we only knocked-down 4EBP1 in our study; depletion of 4EBP1 plus its homolog 4EBP2 may enhance the effects observed here, as was seen in another context^40^, and further cooperate with eEF2K inhibition.

LC–MS/MS analysis uncovered proteins whose steady state levels were significantly induced or suppressed following knockdown of eEF2K, 4EBP1 or both. These proteins may be directly regulated by eEF2K and/or 4EBP1 or are downstream targets of such factors. Regardless of the mechanism, these proteins are stably elevated following eEF2K and/or 4EBP1 inhibition and shape the fate of these cells. Overall, we identified effects on three major groups of proteins that are most influenced by eEF2K and 4EBP1: (1) induction of CDK1 and CDK6 as well as the previously identified and herein verified, Cyclin D1 and c-MYC. These factors promote cell cycle progression under permissive conditions, but may also induce apoptosis under nutrient deprivation or lack of survival factors. Indeed, diverse types of TNBC cells have been shown to be highly sensitive to both activation and inhibition of the CHK2/CDC25/WEE1 pathway, which regulates CDK1 phosphorylation and the G2/M transition^41,42^. (2) Induction of ‘Collagen containing extracellular matrix’, various collagens and regulators that promote cell migration; and (3) suppression of IFN-dependent immune response.

Translational regulation plays a pivotal role in cancer metastasis^43^. Here we showed that depletion of both eEF2K and 4EBP1 increased levels of proteins implicated in cell migration and invasion, suggesting that these two factors may mediate upstream signalling pathways that promote dissemination during normal homeostasis and cancer. eEF2K is regulated by mTORC1, MEK/ERK-p90^RSK^, Ca2^+^/calmodulin-dependent protein kinase, and AMPK, which are often dysregulated in cancer^44^. 4EBP1 is regulated by phosphorylation by mTORC1 but is also induced at the mRNA level in cancer^22^.

Our results raise several questions denoted a-e in Fig. 7. (a) Would combined deletion of 4EBP1 and 4EBP2 further increase the effect seen by 4EBP1 depletion alone on the proteome or lead to induction of additional/unique targets? (b) What’s the basis for our observation that combined depletion of 4EBP1 plus eEF2K induces overlapping proteins? Proteins induced in response to 4EBP1 and/or eEF2K depletion may represent the proteome in the cell, or a subset of proteins that is specifically kept in check by 4EBP1 and eEF2K. Alternatively, a subset of protein translation initiation and elongation may be coordinately regulated. (c) Depletion of 4EBP1, eEF2K or both factors induced the Leucine transporter SLC7A5/LAT1, suggesting an auto-regulatory feed-forward loop in which SLC7A5/LAT1 increases large neutral amino acids (mainly Leu) uptake, leading to mTORC1 induction, which in turn suppresses eEF2K and 4EBP1 thereby stimulating cap-dependent protein translation of SLC7A5/LAT1. A reciprocal induction of SLC7A5/LAT1 and mTORC1 has previously been suggested, but the mechanism is not fully understood. Our results suggest at least one mechanism whereby Leu availability and translocation induces mTORC1, leading to protein translation of SLC7A5/LAT1 and further Leu transport.

Finally, (d–e) what mechanism(s) account for the inhibition of cell growth in response to inhibition of eEF2K and 4EBP1? In one scenario (d), disruption of these protein translation factors induces pathways that are not compatible with each other, leading to cell growth arrest/death that is enhanced under nutrient deprivation. Indeed, eEF2K depletion induces proteins such as CDK1 that promote cell proliferation. However, under Gln starvation, this increase in cell cycle regulators likely kills tumor cells, by enforcing cell proliferation in the absence of sufficient nutrients. Alternatively (e), the cause of cell cycle arrest/death is excessive protein translation, which reduces cellular ATP and induces metabolic crisis under nutrient deprivation. Possibly, both scenarios impact cell survival, and the dominant pathway is context-specific and tumor-dependent.

Our results should inspire efforts to generate clinically relevant, potent and specific eEF2K inhibitors. Notably, the inhibitor we used in this study, TX1918^11^, is relatively specific, but, as previously noted, contains a reactive side chain that is predicted to interact with glutathione in the blood, diminishing its half-life^6^. Several other eEF2K inhibitors have been proposed including cefatrizine^45^ but their specificity and clinical utility have not been established. Targeted protein degradation^46^ or allosteric^47^ rather than kinase inhibitors may prove more effective in specifically targeting this unique calcium/calmodulin-dependent protein kinase. Such inhibitors can be tested in vitro as described herein, and then in vivo using immune-competent mouse models of TNBC^6,48,49^ and human TNBC-derived xenografts. Importantly, *eEf2k* null mice are viable and phenotypically normal^50^, suggesting eEF2K is an ideal target for therapy as its inhibition would likely have minimal adverse effects in most tissues.

## Methods

### Cell lines and cultures

Human breast cancer cell lines, BT-549, Hs578t, MCF7, MDA-MB-231, MDA-MB-436, and MDA-MB-468, were maintained in DMEM containing 10% FBS and 1% penicillin–streptomycin (PEST), at 37 °C with 5% CO_2_. BT549, MDA-MB-436, and MDA-MB-231 were kindly obtained from the late Dr. Mona Gauthier. The remaining lines were purchased from the American Type Culture Collection (ATCC).

### MTT viability and ATP assays

Cells were seeded in 96-well plates at 2–3 × 10^3^ cells/well and treated following day. After three days, 30 µl of 2 mg/ml of MTT (3-[4,5-dimethylthiazol-2-yl]-2,5-diphenyl tetrazolium bromide, Sigma) was added into each well and plates were incubated at 37 °C for 3 h. MTT/media was removed prior to adding 100 µl DMSO. Optical density (OD) was measured at 570 nm by a 96-well microplate reader (Molecular Devices). Assays were performed in 3–6 replica and repeated at least 3 times. In some experiments cell growth was assessed by MTT viability assay and hemocytometer cell counting performed each day during the 4-day period. For ATP analysis, cells were treated with DOX for 4 days, collected and seeded (1 × 10^4^ cells) onto 96 well-plates. Relative ATP levels were determined using the CellTiter-Glo Luminescent Cell Viability Assay (G7570, Promega).

### Transfection conditions for Small Interfering RNA (siRNA) and Generation of tet-inducible shRNA cell lines

siRNA transfection reagent, and Opti-MEM reduced-serum transfection medium were purchased from Dharmacon. A day before transfection, 1–2 × 10^4^ cells were seeded in each of 96-well plates with regular medium and incubated for 24 h. Transfection complexes were prepared with eEF2K (L-004950–00-0010) and/or 4EBP1 (L-003005-00-0010) siRNA (ON-TARGETplus Human siRNA, SMARTPool, Dharmacon) and Lipofectamine RNAiMAX Reagent, diluted in Opti-MEM. After 20 min of incubation at room temperature, 50 µl of siRNA-lipofectamine complexes was added directly to the adherent cells, which were pre-washed in PBS. Six hours later, fresh regular medium was added. A scrambled siRNA (Dharmacon) was used as negative control. The cells were incubated at 37 °C 3 days before analysis. For packaging lentiviruses, HEK293T cells at 1–2 × 10^6^ cells were seeded onto 10 cm culture plates. The following day, TRIPZ eEF2K inducible shRNA (RHS4741-EG29904, Dharmacon) and TRIPZ EIF4EBP1 inducible shRNA (RHS4740-EG1978, Dharmacon) were transfected, and viral media were collected 48 h later. Breast cancer cells (1–2 × 10^6^) were plated and incubated with viral media at 37 °C for 48 h. Transfected clones were selected for 2 weeks in 3 μg/ml puromycin-containing media. A TRIPZ empty shRNA (Dharmacon) was used as negative control. Clones were then screened for efficient depletion of eEF2K expression upon addition of Doxycyline. We then further transduced these cells with lentivirus expressing tet-inducible shRNA for 4EBP1 as well as a lenti-virus expression GFP followed by cell sorting for GFP+ cells.

### Western blot (WB) analysis

For coomassie blue staining, 1 × 10^4^ of cells were collected and lysed in 100 µl of lysis buffer. 10 µl of lysates were loaded. For western blots, 20 µg of total proteins were loaded. All primary antibodies were used at 1:500 dilution: rabbit anti-eEF2K (#3692, Cell Signaling Technology), rabbit anti-phospho-eEF2 (Thr56) (#2331, Cell Signaling Technology), rabbit anti-Cyclin D1 (#2922, Cell Signaling Technology), 4EBP1, p-dIF4E, rabbit anti-Tubulin (#2148, Cell Signaling Technology), rabbit anti-Collagen Type I Alpha 1 (COL1a1) (PAA350HU02, Cloud Clone Corp.), mouse anti-C-myc (9E10) (generously provided by Dr. Linda Penn), mouse anti-CDC2 (CDK1) (#9116, Cell Signaling Technology), and mouse anti-Actin (JLA20, Developmental Studies Hybridoma Bank) antibodies.

### LC–MS/MS analysis

Cell samples were reduced in 10 mM TCEP, 60 °C, 1 h, alkylated in 18 mM iodoacetamide, RT, 30 min in the dark. Precipitated in 1 ml of cold acetone, − 20C, 1 h, resuspended in 50 mM TEAB (triethyl ammonium bicarbonate) and lysed in Trypsin/LysC (Promega), 1.5 ug per sample, 1:50–1:100, 37 °C, overnight. After lyophilisation, 50 μg of proteins was labeled with 10-plex TMT reagents according to the manufacturer’s instructions (90,110, Thermo Fisher Scientific) for 1 h shaking. Excess TMT label was quenched with 8% ammonium hydroxide before TMT-labeled samples were pooled at a one-to-one ratio, vacuum-centrifuged (Speedvac), and stored at − 80 °C. Lyophilized TMT mixes were resuspended in 20 μL of ddH2O and fractionated using high-pH reverse phase high-pressure liquid chromatography (HPLC) at 4 °C^51^. The 60 high-pH fractions were lyophilized, resuspended in 100 μl of 0.1% formic acid, and transferred to a 96-well plate. Each fraction was loaded in its entirety onto a disposable Evotip C18 trap column (Evosep Biosystems, Denmark) as per the manufacturer’s instructions, as described^39^, and analyzed by (Orbitrap Fusion Lumos Tribrid mass spectrometer (Thermo Fisher Scientific, USA). Protein identification and quantification was analyzed using Software Proteome Discoverer v2.2 (Sequest HT), and the database was searched against Uniprot-Human-Jan152018.fasta.

The MS results were normalized for the total peptide amount as following. We calculated peptide abundances as the sum of all reporter ion abundances. The search algorithms then normalized the peptide groups and protein abundances and scaled them. The human database searched for this study contained 48,177 sequences. The LC–MS/MS data were deposited to MassIVE database and are accessible via FTP access: ftp://massive.ucsd.edu/MSV000086059/.

### Volcano plots and G-profiler

While number of peptides with accessions was 3461, only 2394 accessions had complete information for the Volcano analysis. Differential abundances of the normalized proteins (on a log2 scale) were calculated using the package limma^52^ for the statistical software R (https://cran.r-project.org). This software uses a t-test to estimate differences between groups, and “borrows” information across multiple proteins to produce a better estimate of the standard deviation. Each of the 2394 accessions was analyzed, and the *P* values from each test were corrected for multiple testing using Benjamini & Hochberg false discovery rate (FDR).

For pathway analysis, protein ID lists (over 0.5 or below − 0.2 Log 2FC) were downloaded into G-profiler (https://biit.cs.ut.ee/gprofiler/gost) and run as multi_query using Bonferroni correction and a user threshold of 0.05.

### Statistics

Comparison of 2 means was performed using a 2-tailed Student’s t-test. Multiple sample comparisons were calculated using ANOVA, followed by Tukey’s post-hoc analysis.

## Supplementary information

Supplementary Information 1.

Supplementary Information 2.
