# Supplementary material for: Inhibition of eEF2K synergizes with glutaminase inhibitors or 4EBP1 depletion to suppress growth of triple-negative breast cancer cells
Source: Sci Rep. 2021 Apr 28;11:9181. doi: 10.1038/s41598-021-88816-1 (PMC8080725; doi:10.1038/s41598-021-88816-1)
Supplement: Supplementary file 2 — Supplementary Information 2. [file 41598_2021_88816_MOESM2_ESM.pdf]

Inhibition of eEF2K synergizes with Glutaminase inhibitors or 4EBP1 depletion to suppress growth in triple-negative breast cancer cells

YoungJun Ju[1], Yaacov Ben-David[2], Daniela Rotin[3] and Eldad Zacksenhaus[1,*]

LEGENDS TO SUPPLEMENARY DATA

Supplementary Fig. S1. (**A**) Detailed experiments for the calculation of IC50 for the eEF2K inhibitor TX1918 in the indicated breast cancer cell lines. (**B**) Western blots showing the level of depletion of eEF2K and 4EBP1 in tet-inducible shRNA Hs578t cells with two shRNAs (#1, #2) for eEF2K and one for 4EBP1 (#3). (**C-D**) Effect of eEF2K and/or 4EBP1 knockdown on cell growth measured by MTT assay versus cell counting by trypan blue staining. Difference in curve fitting between DOX induced Double knockdown (eEF2K plus 4EBP1) vs no DOX shows significance in both assays but to different extent; * $p < 0.05$; *** $p < 0.0001$. (**E**) Relative cellular ATP in DOX induced eEF2K and/or 4EBP1 shRNA-depleted Hs578t cells. n=3, * $p < 0.05$, ** $p < 0.01$ vs empty vector.

Supplementary Fig. S2. Uncropped images of Western blots shown in this manuscript.

Supplementary Fig. S3. Genomic alterations and impact on survival of CDK1 and COL1A1 in BC. Mutation frequency of CDK1 (**A**) and COL1A1 (**B**) in breast cancer using cBioPortal (combined 9112 patients; 17 studies). Kaplan-Meier survival curves of CDK1 and COL1A1 as function of their mRNA level in BC (**C**) and TNBC (**D**).

Supplementary Fig. S4. LC-MS/MS-based identification of proteins that are down-regulated following eEF2K and/or 4EBP1 knockdown. (**A**) Lists of 20 most down-regulated proteins, such as Interferon-induced GTP-binding protein Mx2, following

eEF2K and/or 4EBP1 knockdown in Hs578t cells. (**B**) Venn diagram of down-regulated proteins.

SUPPLEMENTARY TABLES

Supplementary Tables S1 and S2. Showing primary LC-MS analysis with proteins that are up- or down-regulated following depletion of eEF2K, 4EBP1 or both versus control.

Supplementary Table S3. Showing overlaps between proteins induced 2 fold following eEF2K, 4EBP1 or eEF2K plus 4EBP1 (double).

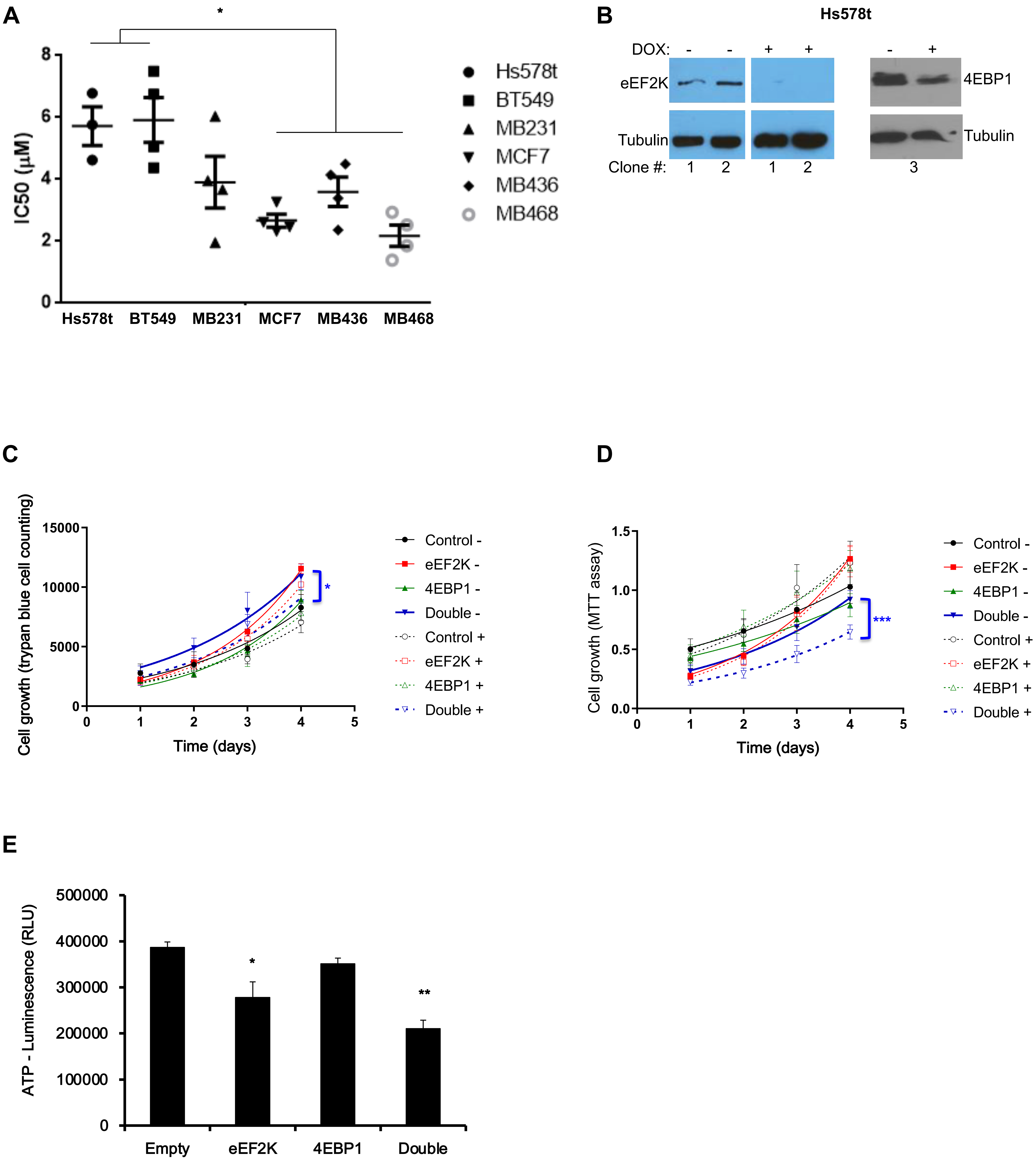

Supplementary Figure 1

Uncropped Western blots

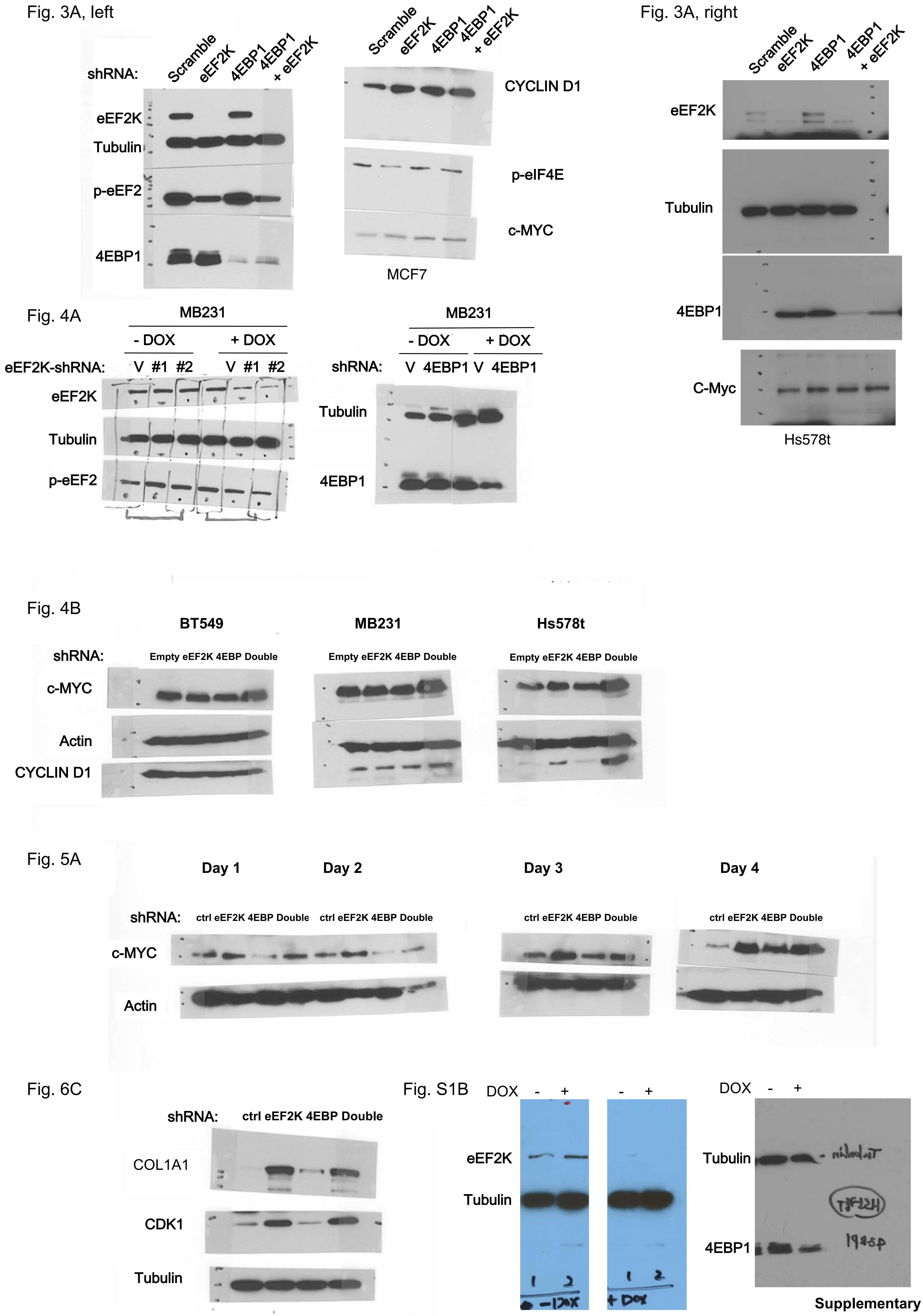

Supplementary Figure 2

A

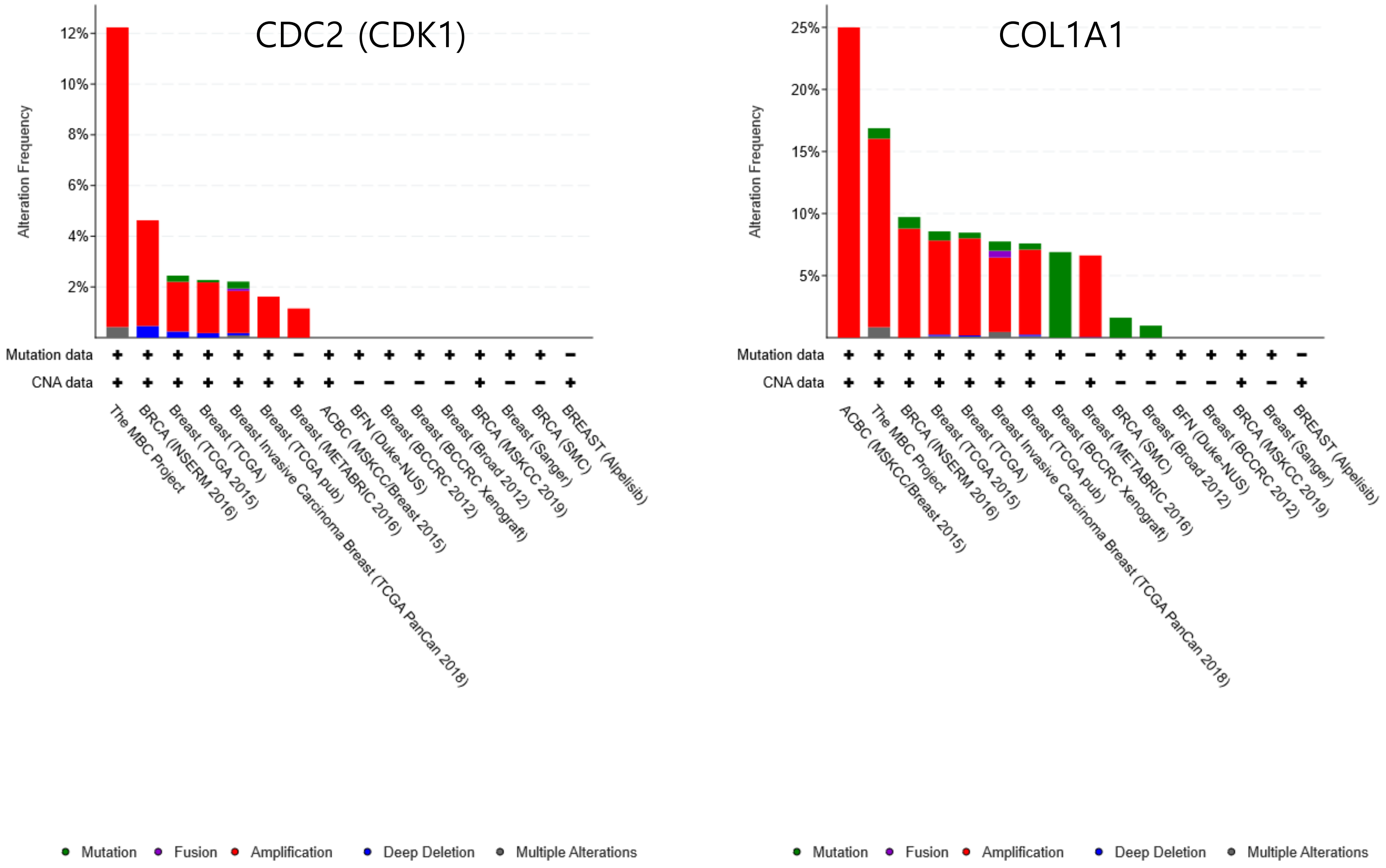

KM plot in BC

B

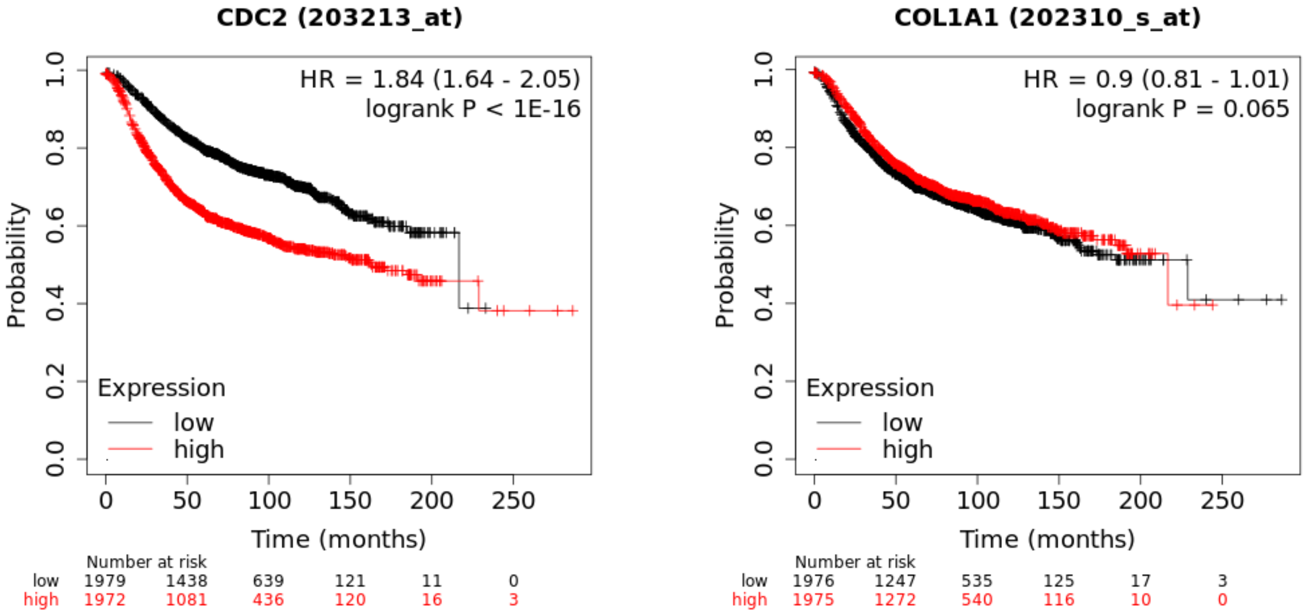

KM plot in TNBC

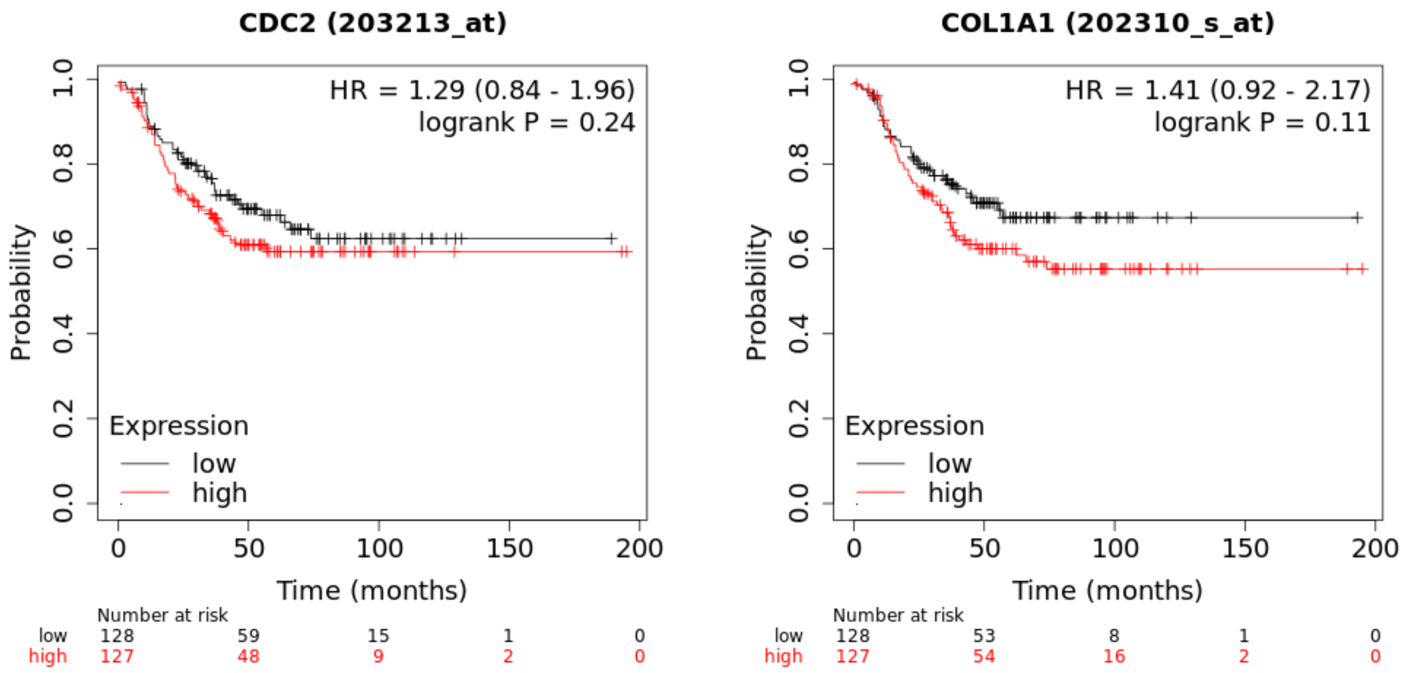

Supplementary Figure 3

A

eEF2K KD

Protein		Decreased
ID	Name	
P20592	Interferon-induced GTP-binding protein Mx2	-0.9316
O14879	Interferon-induced protein with tetratricopeptide repeats 3	-0.89651
Q95604	HLA class I histocompatibility antigen, Cw-17 alpha chain	-0.88022
Q03519	Antigen peptide transporter 2	-0.77148
P09914	Interferon-induced protein with tetratricop eptide repeats 1	-0.75718
Q03518	Antigen peptide transporter 1	-0.75328
P30479	HLA class I histocompatibility antigen, B-4 1 alpha chain	-0.75327
Q10589	Bone marrow stromal antigen 2	-0.69866
O15533-3	Isoform 3 of Tapasin	-0.69172
O95786	Probable ATP-dependent RNA helicase D DX58	-0.6862
P42224	Signal transducer and activator of transcription 1-alpha/beta	-0.6565
Q8TDB6	E3 ubiquitin-protein ligase DTX3L	-0.62441
P36551	Oxygen-dependent coproporphyrinogen -III oxidase, mitochondrial	-0.6149
P32455	Guanylate-binding protein 1	-0.59514
Q06323-2	Isoform 2 of Proteasome activator complex subunit 1	-0.58654
Q16644	MAP kinase-activated protein kinase 3	-0.58621
P00338-3	Isoform 3 of L-lactate dehydrogenase A chain	-0.58022
P08237-3	Isoform 3 of ATP-dependent 6-phospho-fructokinase, muscle type	-0.5779
O96005	Cleft lip and palate transmembrane protein 1	-0.57635
O95678	Keratin, type II cytoskeletal 75	-0.55226

4EBP1 KD

Protein		Decreased
ID	Name	
Q15276	Rab GTPase-binding effector protein 1	-0.5122
Q96R06	Sperm-associated antigen 5	-0.47679
Q9UPZ6	Thrombospondin type-1 domain-co ntaining protein 7A	-0.46603
O75131	Copine-3	-0.46296
P20592	Interferon-induced GTP-binding protein Mx2	-0.44438
Q9NQA3	WAS protein family homolog 6	-0.41867
O95678	Keratin, type II cytoskeletal 75	-0.41551
P09914	Interferon-induced protein with tetra tricopeptide repeats 1	-0.40271
P25440-2	Isoform 2 of Bromodomain-containing protein 2	-0.39103
Q8NHU6	Tudor domain-containing protein 7	-0.39005
P14902	Indoleamine 2,3-dioxygenase 1	-0.38362
Q10589	Bone marrow stromal antigen 2	-0.37011
Q53LP3	Ankyrin repeat domain-containing p rotein SOWAHC	-0.35965
O95025	Semaphorin-3D	-0.35574
Q9UP95-7	Isoform 7 of Solute carrier family 12 member 4	-0.3528
Q96L93-2	Isoform 2 of Kinesin-like protein KIF16B	-0.34914
P48060	Glioma pathogenesis-related protein 1	-0.34804
P62328	Thymosin beta-4	-0.34205
P31153	S-adenosylmethionine synthase isoform type-2	-0.33333
Q7Z5J4	Retinoic acid-induced protein 1	-0.32869

Double KD

Protein		Decreased
ID	Name	
P20592	Interferon-induced GTP-binding protein Mx2	-0.94005
O14879	Interferon-induced protein with tetratrico-peptide repeats 3	-0.87256
Q95604	HLA class I histocompatibility antigen, Cw-17 alpha chain	-0.83697
Q03519	Antigen peptide transporter 2	-0.75964
Q03518	Antigen peptide transporter 1	-0.73821
P09914	Interferon-induced protein with tetratrico-peptide repeats 1	-0.73767
P30479	HLA class I histocompatibility antigen, B-41 alpha chain	-0.73477
Q10589	Bone marrow stromal antigen 2	-0.73018
O15533-3	Isoform 3 of Tapasin	-0.67957
P42224	Signal transducer and activator of transcription 1-alpha/beta	-0.65008
Q8TDB6	E3 ubiquitin-protein ligase DTX3L	-0.64981
O95786	Probable ATP-dependent RNA helicase DDX58	-0.6404
Q06323-2	Isoform 2 of Proteasome activator complex subunit 1	-0.60695
P48060	Glioma pathogenesis-related protein 1	-0.60294
P00338-3	Isoform 3 of L-lactate dehydrogenase A chain	-0.59189
Q9UL46	Proteasome activator complex subunit 2	-0.58045
Q92609-2	Isoform 2 of TBC1 domain family member 5	-0.57823
P36551	Oxygen-dependent coproporphyrinogen-III oxidase, mitochondrial	-0.57523
Q9BYK8	Helicase with zinc finger domain 2	-0.5662
P32455	Guanylate-binding protein 1	-0.55226

B

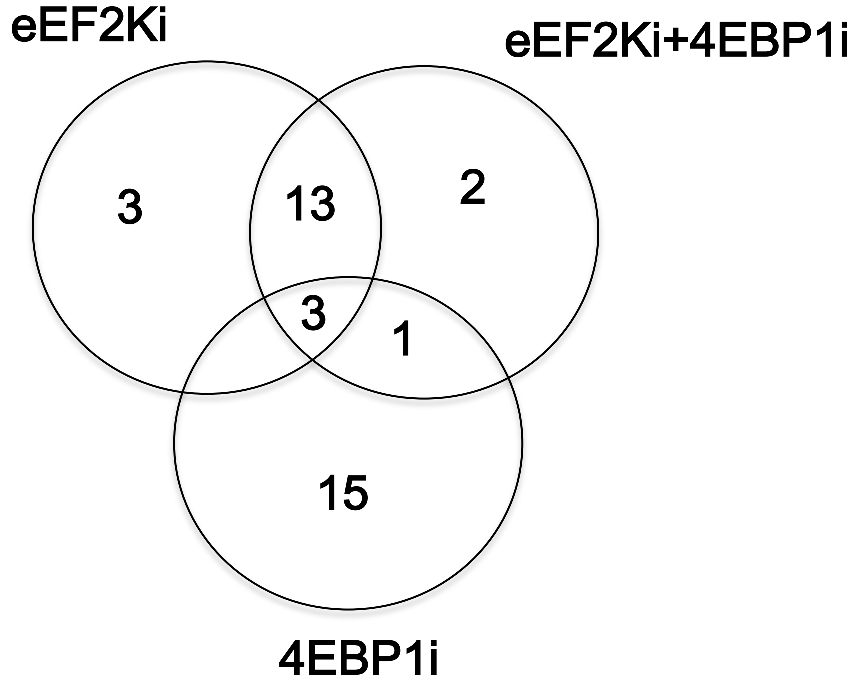

Supplementary Figure 4
